# Different methods of eubiotic feed additive provision affect the health, performance, fermentation, and metabolic status of dairy calves during the preweaning period

**DOI:** 10.1186/s12917-022-03239-y

**Published:** 2022-04-12

**Authors:** Barbara Stefańska, Frank Katzer, Barbara Golińska, Patrycja Sobolewska, Sebastian Smulski, Andrzej Frankiewicz, Włodzimierz Nowak

**Affiliations:** 1 Department of Grassland and Natural Landscape Sciences, Poznań University of Life Sciences, Poznań, Poland; 2 Department of Disease Control, Moredun Research Institute, Penicuik, UK; 3 Department of Internal Diseases and Diagnostics, Poznań University of Life Sciences, Poznań, Poland; 4 Department of Animal Nutrition, Poznań University of Life Sciences, Poznań, Poland

**Keywords:** Calf rearing, Probiotic, Phytobiotic, Rosmarinic acid, Feed additive, Diarrhea

## Abstract

**Background:**

This study aimed to evaluate whether different methods of providing eubiotic feed additives to neonatal calves, during the preweaning period, can improve the calves’ health, performance, ruminal fermentation, and metabolic status. Forty-four (3-day-old) Holstein–Friesian dairy calves (22 female and 22 male) were divided into four treatment groups for the duration of the 8-week trial. The eubiotic feed additive consisted of a combination of probiotic *Lactobacillus* spp. (multiple-strains at a dose of 250 mg/calf/day) and phytobiotics containing rosmarinic acid, as the main bioactive compound (at a dose of 50 mg/calf/day). The groups were named: CON (control, without eubiotic in either the milk replacer or the starter feed), MR (eubiotic in the milk replacer), SF (eubiotic in the starter feed), MRS (eubiotic in both the milk replacer and the starter feed). The individual intake of starter feed and the fecal scores were measured daily, and body weight and biometric measurements were taken weekly until calves were 56 days of age. Blood samples were collected on day 3 and then every 14 days to determine concentrations of insulin-like-growth-factor-I, β-hydroxybutyrate, non-esterified fatty acids, and blood urea nitrogen. Ruminal fluid was collected on days 28 and 56 for short-chain fatty acids, NH_3_-N, and pH measurements.

**Results:**

The body weight of the calves of the MR treatment group was higher compared to all other groups on days 28 and 56. Including the eubiotic feed additive in the milk replacer increased average daily gain, starter intake, and total dry matter intake from day 29 to day 56 and the overall experimental period compared to the CON group. The calves with MR treatment had lower fecal scores from days 3 to 28, a number of parasite oocysts/cysts per gram of feces on day 28, and the occurrences of fecal consistency scores of 3 (mild diarrhea) and 4 (severe diarrhea) were 3.2 and 3.0 times lower, respectively, compared with the CON group. The MR group had higher ruminal concentrations of short-chain-fatty-acids, propionate, and butyrate on day 56 than the CON group. Adding eubiotics into milk replacer resulted in the highest concentrations of blood insulin-like-growth-factor-I and β-hydroxybutyrate from days 29 to 56 and the overall experimental period.

**Conclusion:**

The addition of eubiotic feed additives into the milk replacer can improve health, performance, ruminal fermentation, and biochemical blood indices in dairy calves during the preweaning period.

## Background

Early-life nutrition has become a topic of increasing research interest because the production of well-growing dairy calves and heifers is very important for the future economic success of all dairy farms. Therefore, the growth phase between birth and weaning is of major economic importance [1, 2]. Overall, the health status of the pre-weaned dairy calves can greatly affect lifelong production, including growth, reproductive efficiency, and milk production [3]. Calves are at a greater risk of dying during the first 21 days of life than during the rest of the rearing period [4]. This is because calves are normally agammaglobulinemic at birth, which means that they are born without blood IgG [2]. In cattle, maternal IgG is not transferred across the placenta during pregnancy and the newborn calves are only able to produce their own IgG after exposure to pathogens resulting in detectable levels of their own immunoglobulins from approximately 14 days of age. It is known that timely delivery of good quality and quantity of colostrum as well as the rate and amount of absorption of intestinal IgG are the factors that determine the successful passive transfer of immunity in calves [2]. While negative outcomes are associated with the failure of passive immune transfer include and these include increased morbidity and mortality. The incidence of mortality in the perinatal period, defined as the duration from birth to 48 h after birth, ranges in dairy herds worldwide from 3 to 9% [5]. In USA dairy herds, current mortality rates of 5% and morbidity rates of 34% were published for preweaning calves [6]. In addition, diseases during the neonatal stage, especially diarrhea, significantly affect the growth during the rearing period, the long-term future performances, and the loss of genetic potential for future herd improvements [7, 8]. Feeding management during the neonatal and preweaning period greatly impacts the success of calf rearing and, in addition, it affects health and performance in later life [2, 9]. Although mortality in calves is unlikely to be entirely eradicated, reducing it as much as possible should be a goal [10]. Therefore, it is important to stimulate the immunity and health of a neonatal calf by providing feed additives, especially during the critical period of the first weeks of life. Understanding the relationship between management practices, nutritional strategies, and calf health is essential for minimizing morbidity and mortality and enhancing future production. In intensive rearing and management systems, diets have been supplemented widely with antibiotics as feed additives to reduce the impact of infectious diseases associated with diarrhea and respiratory disease. However, public and scientific concern about the use of antibiotics as feed additives in animal production, resulting potentially in antibiotic resistance, environmental contamination, and their presence in foods of animal origin, lead the European Union (EU) to ban the use of antibiotics in livestock as production enhancers on 22^nd^ September 2003 [11]. Therefore, new studies have been undertaken in recent years to develop alternatives to antibiotics, such as natural feed additives, for reducing morbidity and mortality, especially during the first 8 weeks of the rearing period. Considerable evidence exists in the literature of the potential effects of natural feed additives on dairy calves' health, growth performance, and ruminal fermentation. The aims of most of the published research in dairy calf nutrition were to compare the effectiveness of the different types of feed additives, such as probiotics [12–14], prebiotics [15, 16], phytogenic substances [17], essential oils [18] or their blends, especially combinations of essential oils and prebiotics [19, 20]. A recent study incorporated a eubiotic feed additive (consisting of a combination of a 250 mg/calf/day multi-strain probiotic (containing *Lactobacillus casei, Lactobacillus salivarius*, and *Lactobacillus sakei*) and 50 mg/calf/day of herbal extracts (with rosmarinic acid as the main bioactive component)) into the liquid feed (colostrum and milk replacer)of dairy calves [21]. The provision of this feed additive improved the health status (decreasing diarrhea occurrence, *Cryptosporidium* spp., and *Giardia duodenalis* prevalence), feed intake, growth performance, and metabolic status of dairy calves during the preweaning period [21]. Feed additives can be mixed with liquid feeds, such as whole milk or milk replacer, or solid feeds like the calf starter. However, only very limited scientific data are available comparing the effects of administering the feed additives with all these feeds on calves’ health, growth performance, and the metabolic status during the preweaning period [22]. This information would be particularly useful for preventing infectious diseases that cause diarrhea and modeling their effects on the growth of dairy calves, especially during the important preweaning period. Beneficial effects of eubiotic feed additives are already described. Therefore, this study aimed to evaluate whether the different methods of providing eubiotic feed additives can improve calf health and performance, ruminal fermentation, and the metabolic status during the preweaning period of neonatal calves. We hypothesized that providing a eubiotic feed additive in the milk replacer as well as in the starter feed during the period between birth and weaning of dairy calves would have a more beneficial effect than only providing it in the milk replacer or in the starter feed. We expect that this will lead to a decreased occurrence of diarrhea, enhanced growth performance, improved ruminal fermentation, and better blood metabolite levels.

## Results

The treatment groups differed in starter intake, total dry matter intake (TDMI), average daily gain (ADG), and body weight (BW). As expected, calves consumed little solid feed during the first 4 weeks of life (Table 1). Calves consumed more starter intake and TDMI depending on the treatment group during the 29–56 days period (*P* = 0.019 and *P* = 0.028, respectively) and the overall experimental period (*P* = 0.022 and *P* = 0.015, respectively), and the greatest effect was noted in the MR treatment group in comparison to the CON treatment group. Greater BW on days 28 (*P* = 0.022) and 56 (*P* = 0.034) of the experiment was noted in the MR treatment group compared to other experimental groups. In addition, the MR treatment resulted in higher ADG during 29–56 days (*P* = 0.024) and the overall experimental periods (*P* = 0.018). In the current study, calves fed the MR treatment had the lowest fecal scores from 3 to 28 days (*P* = 0.018) compared to the CON and SF groups. The calves of the MR treatment group had fewer parasite oocysts/cysts per gram of feces (EPG) on day 28 (*P* = 0.032) than those of the CON group. In addition, the occurrence of score 3, indicating mild diarrhea, and score 4, confirmation of diarrhea, were respectively 3.2 (*P* = 0.024) and 3.0 (*P* = 0.016) times lower for calves fed MR treatment compared with animals in the CON group, without feed additives, during the entire experimental period (Table 2). Starter intake, TDMI, total crude protein (CP) intake, ADG, and biometric measurements such as changes in body length (BL), hip height (HH), hip-width (HW), heart girth (HG) increased with the age of calves (effect of the period; *P* < 0.001). No effects were detected, dependent on the method of the eubiotic feed additive provision, on milk replacer intake, total CP intake, feed efficiency (FE), and changes in all biometric measurements.

**Table 1:** The effect of different methods of eubiotic feed additive provision on intake and growth performance in dairy calves

Item	Treatment^1^				SEM	*P*-values		
	CON	MR	SF	MRS		Treatment	Period	Treatment x Period
Starter intake (kg/day)								
Period 3—28 days	0.26	0.27	0.22	0.28	0.11	0.46	< 0.001	0.10
Period 29—56 days	0.47^b^	0.78^a^	0.54^ab^	0.59^ab^	0.14	0.019	< 0.001	0.09
Overall 3—56 days	0.37^b^	0.54^a^	0.39^ab^	0.44^ab^	0.12	0.022	< 0.001	0.11
Milk replacer intake (kg/day)								
Period 3—28 days	0.810	0.810	0.810	0.810	0.01	0.62	0.59	0.84
Period 29—56 days	0.680	0.680	0.680	0.680	0.06	0.54	0.79	0.63
Overall 3—56 days	0.740	0.740	0.740	0.740	0.04	0.43	0.56	0.46
TDMI^2^ (kg/day)								
Period 3—28 days	1.07	1.08	1.03	1.09	0.06	0.42	< 0.001	0.24
Period 29—56 days	1.15^b^	1.46^a^	1.22^ab^	1.27^ab^	0.08	0.028	< 0.001	0.22
Overall 3—56 days	1.11^b^	1.28^a^	1.13^ab^	1.18^ab^	0.04	0.015	< 0.001	0.36
Total CP intake (kg/day)								
Period 3—28 days	0.26	0.26	0.25	0.27	0.01	0.32	< 0.001	0.32
Period 29—56 days	0.28	0.35	0.29	0.31	0.04	0.52	< 0.001	0.42
Overall 3—56 days	0.27	0.31	0.27	0.29	0.02	0.35	< 0.001	0.35
Body weight (kg)								
3 day of age	44.5	44.3	44.6	44.4	0.46	0.45	-	-
28 day of age	53.4^b^	55.5^a^	51.9^b^	51.6^b^	0.66	0.022	-	-
56 day of age	70.5^b^	77.5^a^	69.3^b^	69.6^b^	0.87	0.034	-	-
ADG^3^ (kg/day)								
Period 3—28 days	0.36	0.45	0.29	0.29	0.15	0.93	< 0.001	0.97
Period 29—56 days	0.60^b^	0.79^a^	0.62^b^	0.64^b^	0.22	0.024	< 0.001	0.50
Overall 3—56 days	0.49^b^	0.63^a^	0.47^b^	0.48^b^	0.19	0.018	< 0.001	0.95
FE^4^								
Period 3—28 days	0.34	0.46	0.38	0.37	0.10	0.32	0.46	0.10
Period 29—56 days	0.54	0.54	0.50	0.48	0.13	0.83	0.19	0.13
Overall 3—56 days	0.46	0.51	0.45	0.44	0.42	0.32	0.82	0.42
Body length change (cm)								
Period 3—28 days	5.10	5.90	5.90	5.80	1.10	0.19	< 0.001	0.28
Period 29—56 days	7.50	7.30	6.40	6.70	1.30	0.62	< 0.001	0.99
Overall 3—56 days	12.6	13.2	12.3	12.5	1.48	0.22	< 0.001	0.81
Hip height change (cm)								
Period 3—28 days	5.20	4.90	4.10	4.20	0.78	0.25	< 0.001	0.28
Period 29—56 days	5.40	6.00	5.50	4.50	0.84	0.34	< 0.001	0.99
Overall 3—56 days	10.6	10.9	9.60	8.70	0.87	0.11	< 0.001	0.81
Hip width change (cm)								
Period 3—28 days	2.20	1.80	2.2	3.10	0.29	0.38	< 0.001	0.28
Period 29—56 days	3.20	3.40	2.2	1.90	0.27	0.65	< 0.001	0.99
Overall 3—56 days	5.40	5.20	4.4	5.00	0.40	0.47	< 0.001	0.81
Heart girth change (cm)								
Period 3—28 days	6.00	4.10	5.20	6.20	0.59	0.24	< 0.001	0.28
Period 29—56 days	9.40	11.4	8.10	7.20	0.95	0.16	< 0.001	0.99
Overall 3—56 days	15.4	15.5	13.3	13.4	1.10	0.09	< 0.001	0.81
Fecal score								
Period 3—28 days	1.72^a^	1.10^b^	1.47^a^	1.25^ab^	0.02	0.018	< 0.001	0.342
Period 29—56 days	1.09	1.06	1.06	1.06	0.01	0.32	< 0.001	0.314
Overall 3—56 days	1.16	1.08	1.14	1.09	0.01	0.28	< 0.001	0.344
EPG (× 10^2^/g) ^5^								
3 day of age	0.47	0.45	0.42	0.44	0.01	0.39	-	-
28 day of age	2.12^a^	1.46^b^	1.74^ab^	1.68^ab^	0.03	0.032	-	-
56 day of age	0.86	0.55	0.76	0.65	0.01	0.25	-	-

^1^*Treatment CON* (control: without eubiotic feed additive in their milk replacer or their starter feed: *n* = 11), *MR* (eubiotic feed additive added to their milk replacer: *n* = 11), *SF* (eubiotic feed additive added to their starter feed: *n* = 11), *MRS* (eubiotic feed additive added to their milk replacer and their starter feed: *n* = 11)

^2^*TDMI* total dry matter intake from the milk replacer and the starter feed (kg/day)

^3^*ADG* average daily gain (kg/day) in period 3–28 days = (((weaning BW—initial BW)/25 days); in period 29–56 days = (((final BW—weaning BW)/28 days); during the complete study period = (((final BW—initial BW)/53 days)

^4^*FE* feed efficiency expressed as ADG (kg/day) to TDMI (kg/day) ratio

^5^ *EPG* number of parasite oocysts/cysts per gram of feces, ^a–b^ Means within a column with different superscripts differ significantly (*P* ≤ 0.05)

**Table 2:** The effect of different methods of eubiotic feed additive provision on the occurrences of diarrhea by dairy calves

Item	Treatment^1^				SEM	*P*-values
	CON	MR	SF	MRS		
Diarrhea levels						
Score 1 (times)	42.7^b^	49.9^a^	45.2^ab^	48.8^a^	0.22	0.025
Score 2 (times)	2.80^a^	1.80^b^	2.50^ab^	2.40^ab^	0.07	0.012
Score 3 (times)	3.85^a^	0.65^b^	2.70^ab^	0.85^b^	0.04	0.024
Score 4 (times)	3.65^a^	0.65^b^	2.60^ab^	0.85^b^	0.01	0.016

^1^*Treatment CON* (control: without eubiotic feed additive in their milk replacer or their starter feed: *n* = 11), *MR* (eubiotic feed additive added to their milk replacer: *n* = 11), *SF* (eubiotic feed additive added to their starter feed: *n* = 11), *MRS* (eubiotic feed additive added to their milk replacer and their starter feed: *n* = 11), ^a–b^ Means within a column with different superscripts differ (*P* ≤ 0.05)

The different eubiotic feed additive provision methods did not affect ruminal fluid pH (Table 3). The ruminal fluid of the treatment groups differed in the total short-chain fatty acids (SCFA) detectable (*P* = 0.012) and molar concentrations of propionate (*P* = 0.037) and butyrate (*P* = 0.022). The MR treatment group had the highest levels of these indices. No relationships were detected between the different methods of eubiotic feed additive provision on concentrations of acetate, n-valerate, ratios of acetate to propionate (C_2_:C_3_), butyrate to valerate (C_4_:C_5_) ratios, and N-NH_3_.

**Table 3:** The effect of different methods of eubiotic feed additive provision on ruminal fermentation in dairy calves

Item	Time (day)^2^	Treatment^1^				SEM	*P*-values
		CON	MR	SF	MRS		
Ruminal pH	28	5.29	5.72	5.71	5.59	0.02	0.49
	56	6.04	6.20	6.44	6.21	0.08	0.53
SCFA^3^ molar concentrations (mmol/L)							
Total SCFA	28	39.8	37.4	38.2	36.6	0.20	0.19
	56	70.6^b^	79.2^a^	75.3^ab^	76.5^ab^	0.11	0.012
Acetate	28	21.9	19.2	21.4	19.4	0.11	0.25
	56	32.2	32.9	35.7	34.5	0.09	0.25
Propionate	28	12.8	12.4	11.2	11.7	0.24	0.30
	56	27.7^b^	32.2^a^	27.3^b^	29.3^ab^	0.66	0.037
N-butyrate	28	3.76	4.70	4.35	4.26	0.06	0.19
	56	7.95^b^	10.9^a^	9.28^ab^	9.48^ab^	0.04	0.022
N-valerate	28	1.38	1.07	1.26	1.20	0.01	0.34
	56	2.75	3.18	3.06	3.22	0.04	0.25
C_2_: C_3_ ratio^4^	28	1.71	1.64	1.73	1.73	0.08	0.08
	56	1.16	1.12	1.11	1.26	0.02	0.25
C_4_: C_5_ ratio^5^	28	2.72	4.39	3.45	3.55	0.06	0.09
	56	2.89	3.42	3.03	2.94	0.06	0.42
NH_3_-N (mmol/L)	28	21.2	13.2	19.5	17.5	1.38	0.16
	56	14.3	12.4	16.2	15.1	1.60	0.12

^1^*Treatment CON* (control: without eubiotic feed additive in their milk replacer or their starter feed: *n* = 11), *MR* (eubiotic feed additive added to their milk replacer: *n* = 11), *SF* (eubiotic feed additive added to their starter feed: *n* = 11), *MRS* (eubiotic feed additive added to their milk replacer and their starter feed: *n* = 11)

^2^*Time* age of calf (day)

^3^ *SCFA* short-chain fatty acids

^4^*C*_*2*_*: C*_*3*_* ratio* the ratio of ruminal acetate to propionate

^5^ *C*_*4*_*: C*_*5*_* ratio* the ratio of ruminal butyrate to valerate, ^a–b^ Means within a column with different superscripts differ (*P* ≤ 0.05)

All the biochemical blood analyses results were affected by the age of the calves (*P* = 0.01; Table 4). Higher concentrations of insulin-like growth factor-I (IGF-I) and *β*-hydroxybutyrate (BHBA) were detected in the MR treatment group, during the 29–56 days (*P* = 0.028 and *P* = 0.033, respectively) period and the overall experimental period (*P* = 0.022 and *P* = 0.028, respectively).

**Table 4:** The effect of different methods of eubiotic feed additive provision on biochemical blood indices in dairy calves

Item	Treatment^1^				SEM	*P*-values		
	CON	MR	SF	MRS		Treatment	Period	Treatment x Period
Insulin-like growth factor-I (ng/mL)								
Period 3—28 days	43.9	44.1	38.8	42.4	2.65	0.25	< 0.001	0.45
Period 29—56 days	37.7^b^	53.4^a^	39.2^b^	39.3^b^	2.21	0.028	< 0.001	0.56
Overall 3—56 days	41.4^b^	51.0^a^	38.4^b^	41.2^b^	2.88	0.022	< 0.001	0.59
β-hydroxybutyrate (mmol/L)								
Period 3—28 days	0.40	0.39	0.39	0.40	0.01	0.25	< 0.001	0.22
Period 29—56 days	0.51^b^	0.62^a^	0.50^b^	0.53^b^	0.01	0.033	< 0.001	0.23
Overall 3—56 days	0.45^b^	0.53^a^	0.45^b^	0.45^b^	0.01	0.028	< 0.001	0.25
Non-esterified fatty acids (mmol/L)								
Period 3—28 days	0.28	0.35	0.36	0.31	0.01	0.59	< 0.001	0.82
Period 29—56 days	0.30	0.31	0.38	0.30	0.01	0.60	< 0.001	0.62
Overall 3—56 days	0.29	0.33	0.37	0.31	0.01	0.59	< 0.001	0.51
Blood urea nitrogen (mg/dL)								
Period 3—28 days	8.43	8.43	8.30	8.20	0.22	0.63	< 0.001	0.28
Period 29—56 days	9.99	11.3	11.2	11.2	0.23	0.63	< 0.001	0.32
Overall 3—56 days	9.05	9.56	9.46	9.56	0.28	0.49	< 0.001	0.42

^1^*Treatment CON* (control: without eubiotic feed additive in their milk replacer or their starter feed: *n* = 11), *MR* (eubiotic feed additive added to their milk replacer: *n* = 11), *SF* (eubiotic feed additive added to their starter feed: *n* = 11), *MRS* (eubiotic feed additive added to their milk replacer and their starter feed: *n* = 11), ^a–b^ Means within a column with different superscripts differ (*P* ≤ 0.05)

## Discussion

Considerable evidence of the potential effect of different kinds of natural feed additives [12, 15, 17, 18, 20] on the health, growth performance, ruminal fermentation, and biochemical blood indices of pre-weaned dairy calves is available in the literature. However, currently, only limited published scientific results are available to compare these indicators.

The current study incorporated eubiotic feed additives in the milk replacer and starter feed, which improved calf health during the preweaning period. This was demonstrated by fewer occurrences of diarrhea (scores 3 and 4), lower fecal scores, and coproparasitological indices. Differences between groups were seen in the starter intake uptake and TDMI; in addition, calves supplied with the eubiotic feed additive in their milk replacer (MR treatment group) showed the greatest effects. During the preweaning period, calves of the MR treatment group consumed on average greater amounts of starter intake (0.54 vs. 0.37 kg/day) and TDMI (1.28 vs. 1.11 kg/day) in comparison to the CON group. Also, the calves in the MR group weighed on average 7.0 kg more at the end of the study than the calves of the CON group (without eubiotic feed additive), and the MR calves gained on average 0.14 kg/day weight during the preweaning period. Previous studies have shown similar results to the ones seen in this study. The previous studies showed lower fecal scores and fewer occurrences of diarrhea [21, 22] as well as greater amounts of starter intake [0.56 vs. 0.38 kg/day; 21], TDMI [1.34 vs. 1.20; 21]. They also observed body weight increases and ADG [respectively, 78.5 vs. 77.6 kg and 626 vs. 610 g/day, respectively; [14]] during the rearing period when natural feed additives (probiotics, phytobiotic, or their combination) were incorporated into the milk replacer for the calves [21, 22]. The current results indicate, similarly to our previous results, that feeding calves a milk replacer containing eubiotics with probiotics *Lactobacillus* spp. and the main bioactive compounds (consisting of rosmarinic acid) may enhance the growth performance, feed intake, and the health of calves. The growth performance of young calves is strongly dependent to the type of feed they consume, the rearing system, and the intestinal microbiota balance [16]. Probiotics and essential oils may prevent intestinal microbial imbalances, which are common in an intensive rearing system, to reduce the disease incidence [13, 18, 23]. If calves become ill during the first few weeks of life, growth may decrease and result in death or poor productivity, even after they become adults [2, 24]. It is known that gut bacteria such as *Lactobacillus* spp. and *Bifidobacterium* spp., and *Faecalibacterium* spp. can modulate the immune system and inflammatory response, leading to alterations in metabolism, which can influence feed intake, nutrient utilization, and growth performance [25]. Phytobiotics, such as herbal extracts of *Thymus vulgaris* and *Oregano vulgaris*, contain bioactive essential oils such as phenols (thymol, carvacrol, rosmarinic acid). These bioactive compounds have broad antimicrobial activity, particularly against gram-positive bacteria, by disrupting the bacterial cells membrane [26], leading to improved nutrient digestion by the calves [27]. Different mechanisms of action of probiotics and essential oils, including rosmarinic acid, have been described [12, 16, 18]. Probiotics compete for nutrients and produce antibacterial compounds (e.g., SCFA, hydrogen peroxide, nitric oxide, and bacteriocins) in the intestinal lumen allowing them to occupy specific niches of the intestinal mucosa and activate the innate immune system of calves [28]. Also, rosmarinic acid has antioxidant, antimicrobial (including bacteria, protozoa, and fungi), anti-inflammatory activities, and it can stimulate the endocrine and immune system [29]. The improvement of each of these mechanisms can result in better calf health, feed intake, and nutrient utilization, leading to improved BW and ADG. In some studies, higher ADG was observed in calves that received probiotics and essential oils, mainly in the first two to three weeks of age [14, 20]. It is possible that in the current study, the eubiotic feed additive within the milk replacer affected the calves so that they can faster respond to the stress of the first weeks of life when they are starting to produce immunoglobulins in response to environmental stimulants. It can take around 10 – 14 days until their first immunoglobulins appear [30]. The natural feed additives within the eubiotics support the immune system during this critical period, which positively influences dairy calves' health, productive performance, and the metabolic status during the preweaning period. Therefore, mixing probiotic, phytobiotic, or as in the current study, eubiotic (multi-strain probiotic and phytobiotics, with rosmarinic acid as the main bioactive compound) into the milk replacer might be a strategy to reduce pathogenic bacteria in the gut. The main goal is to promote the colonization of protective bacteria in calves during the first week of their life to compete with pathogenic bacteria responsible for gastrointestinal infections that may cause diarrhea [14, 21, 31]. In addition, natural feed additives can stimulate the development of the immune response against pathogenic bacteria and promote beneficial effects to the host by favoring the balance of the intestinal microbiota [31]. In addition, calves fed eubiotics incorporated into the milk replacer consumed more starter intake and TDMI, which may have improved health and metabolic status, absorption of nutrients from the intestines, and faster ruminal function development, which also improved growth performance such as measured by BW and ADG. The method of feed additive provision may have influenced its effectiveness. The calves are fed mainly a milk replacer or whole milk supplemented with the addition of calf starter feed during the preweaning period. Most published studies evaluated the provision of feed additives in the starter feed for dairy calves, to investigate benefits to ruminal development and accelerate growth performance [20, 32, 33]. However, as seen in the current study, the intake of solid feed (calf starter) by the calves in the first 4 weeks of age is small [34–36], and the timing of the occurrence of enteric diseases is mainly in the first 30 days of life [37, 38]. Due to the calf’s limited capability of ingesting large amounts of solid feed in the first days of life, the supplement intake within the starter is very limited during the early stages of the pre-weaning period, and the desired supplementation level may not be achieved until later, which may mask any effects. In addition, the provision of a eubiotic feed additive in the MR was more efficient than in the SF, resulting in its adequate daily intake during the preweaning period. Similar to our SF treatment results, Seifzadeh et al., [23] showed that calves fed eubiotics, consisting of herbal additives and probiotics, mixed into the calf starter feed resulted in a lower intake of this additive in the first month. The starter feed intake in the first month of life was lower than in the second month of life and therefore the effects of the herbal additives on growth performance were only observed in the second month. In the current study, providing eubiotic feed additive to milk replacer and starter feed (MRS treatment) did not influence starter feed intake, growth performance, ruminal fermentation characteristics, and metabolic status. Thus far, no data have been published about the relationship between providing natural feed additives to both dietary feeding methods (milk replacer and starter feed) on the above-analyzed indices. The results are surprising, and we hypothesize that lower starter feed intake during the first month of calf life might mask the effect of the effectiveness of eubiotic feed additive during the complete rearing period. However, eubiotic consumption levels within the starter feed intake were not analyzed, which is a limitation of the current study and may affect the results and may possibly mask effects. The feed additives should be provided in the liquid feed to increase effectiveness, especially in the first 4 weeks of life [22]. A meta-analysis conducted by Frizzo et al. [31] revealed that beneficial effects on calf growth rates were observed when natural feed additives were added to milk replacer rather than to whole milk in the first few weeks of life, resulting in fewer health and nutritional problems. This may be associated with increased calf stress, in response to milk replacers (different chemical and quality characteristics) in comparison to feeding whole milk during the first few weeks of life, which may also predispose animals to nutritional disorders such as diarrhea [31]. Therefore during the first few weeks of the preweaning period, the eubiotics may be mixed into the milk replacer as a strategy to improve health and performance, and thereafter it can be added into the starter feed. However, further investigations into potential causative mechanisms are needed.

The bioactive compounds of essential oils and probiotics have prompted scientists to examine the potential to manipulate ruminal microbial fermentation to improve feed intake and growth performance [39]. The start of ruminal fermentation can be detected at a very young age, and SCFA can be found in the calves' rumen as early as the second week of life [40]. This is confirmed by enzymatic activities of ruminal microbiota (such as fibrolysis, amylolysis, proteolysis, and ureolysis), observed in the rumen from 4 to 10 days of age [40]. In the current study, the provision of eubiotics within the milk replacer affected ruminal fermentation by increased concentrations of total SCFA, propionate, and butyrate at the end of the preweaning period (day 56). In addition, on days 28 and 56 of life, the ruminal concentration of total SCFA was < 50 mmol/l and > 70 mmol/L, respectively, consistent with previous reports [41, 42]. Currently, there is no explanation for the mechanism of the improved ruminal function and ruminal fermentation after the provision of eubiotics *by-pass* the rumen. It cannot be excluded that eubiotics, consisting of rosmarinic acid as the main bioactive compounds and *Lactobacillus* spp. probiotics, acted by other mechanisms. This may involve altering the metabolism of the lower digestive tract, which could indirectly affect ruminal development. This was suggested as the mode of action for other feed additives, such as sodium butyrate [43] or *Yarrowia lipolytica* yeast culture [35]. According to Hassan et al. [44], the functioning of the reticular groove is less efficient as the calf ages, and in older calves, part of the consumed milk can enter the rumen and influence its fermentation. Rosmarinic acid, the main bioactive compound within the eubiotic feed additive, could cause hydrophobicity and disrupt bacterial membranes, increasing liquid permeability and causing a toxic effect for the microorganisms [45]. This activity could result in inhibition of ruminal deamination and methanogenesis, which might affect the decrease in ruminal nitrogen ammonia, methane, acetate concentrations, acetate to propionate ratio, and an increase of the propionate and butyrate concentrations [46], which are important for ruminal papillae development, and especially propionate is used in the gluconeogenesis route. On the other hand, it could have been a consequence of the positive effect of the treatment on the health of the animals, which may have stimulated, especially in older calves, the increase of solid feed intake like starter feed leading to increased ruminal fermentation. Similar to our results, Quigley et al. [47] showed a greater SCFA concentration with greater feed intake and TDMI. Fermentation of calf starter feed increases the ruminal concentration of SCFA, especially propionate and butyrate, which most likely stimulates papillae development in the rumen [41]. The major metabolic pathway of SCFA metabolism in the ruminal epithelium is ketogenesis [48]. The blood BHBA is produced by the metabolism of butyrate during its passage across the ruminal wall and, in consequence, higher levels of it can be used as an indicator of greater metabolic activity of ruminal epithelial cells [49]. Similarly, in the current study, ruminal butyrate and blood BHBA were higher during the experiment at days 28 and 56 and from days 29 to 56 in calves fed milk replacer containing the eubiotic feed additive. Moreover, in the MR treatment group, a greater blood IGF-I concentration was noted. IGF-I is a hormone produced in many tissues throughout the body, mostly in the liver [50]. It is a growth promoter that regulates the proliferation of many cell types, including epithelial cells of the intestine and rumen [51]. IGF-I is thought to be associated with the energy status of the body. In previous research, higher concentrations of this hormone in the serum corresponded with greater nutrient intake, enhanced growth, and higher body weight [51]. It could have been a consequence of the positive effect of the MR treatment on animal growth and metabolic status, as ruminal epithelial cells require an adequate supply of nutrients for their proliferation and differentiation.

## Conclusion

Feeding calves a eubiotic feed additive provided in the milk replacer reduced the gut health challenges (diarrhea) and improved feed intake, growth performance, and enhanced ruminal fermentation of neonatal dairy calves. Feeding eubiotic feed additives in the liquid feed may provide a natural viable alternative to antibiotics to minimalize health challenges while improving calf growth performance. In addition, the feed additives should be mixed into the liquid rather than the solid feed to increase effectiveness, especially in the first 4 weeks of life, due to better daily intake. However, the biological significance of these results needs to be investigated further in larger field trials.

## Methods

### Eubiotic feed additive characteristic

The experimental eubiotic feed additive consisted of a combination of probiotic multi-strain of *Lactobacillus* spp. at a dose of 250 mg/calf/day and a phytobiotic, where the main bioactive compound was rosmarinic acid, at a dose of 50 mg/calf/day. The probiotics consisted of equal ratios of three *Lactobacillus* species: L. *casei*, *L. salivarius,* and *L. sakei* with a total of 10^11^ CFU/g. These strains were isolated from a healthy Holstein–Friesian calf in Poland and were manufactured by Poznan University of Life Sciences, Poland. These strains are patented with the following Genebank accession numbers: PKM B/00103, PKM B/00102, PKM B/00101. Further details about these strains have been published previously by Stefanska et al., [20]. The phytobiotic additive was prepared by the Institute of Natural Fibers and Medicinal Plants at the National Research Institute, Poznań, Poland and consisted of a watery extract of dried *Thymus vulgaris* and *Oregano vulgaris* to yield the experimental dose of rosmarinic acid, as the bioactive compound, at the level 50 mg/calf/day. Both products (probiotics and phytobiotics) were supplied as dry powders as a single manufactured lot. The preparation details and experimental dose determination of the eubiotic feed additive were described by Stefanska et al., [21]. The stability of the eubiotic feed additive was assessed weekly, during storage, and was viable during the complete experimental period.

### Animals, treatments, and management

The experiment was carried out in a commercial dairy farm between April and June 2015. This study used 44 (3-day-old; 44.5 ± 0.46) Polish Holstein–Friesian dairy calves. They were selected depending on sex (22 male and 22 female calves) and dam parity (22 each born from multiparous and primiparous cows) and assigned randomly into the four treatments groups consisting of 11 calves each for the duration of the study (56 days). The treatment groups differed by the method of the eubiotic feed additive was provided. The groups were: CON (control, without eubiotic feed additive in either milk replacer or starter feed), MR (eubiotic feed additive consisting of a combination of multi-strain probiotic at a dose of 250 mg/calf/day, and a phytobiotic at the dose of 50 mg/calf/day added to the milk replacer), SF (eubiotic feed additive consisted of a combination of multi-strain probiotic at a dose of 250 mg/calf/day and phytobiotic at a dose of 50 mg/calf/day added to the starter feed), MRS (eubiotic feed additive consisted of a combination of the multi-strain probiotic at a dose of 125 mg/calf/day and phytobiotic at a dose of 25 mg/calf/day added to the milk replacer and the same dose added to the starter feed). The eubiotic feed additive, supplied as a dry powder, was mixed into the milk replacer immediately before morning feeding. For the pelleted starter feed, the eubiotic feed additive was blended into the mineral and vitamin premix (50 g/calf/day) and then top-dressed to the starter feed immediately before morning feeding. The calves, all obtained from a single commercial herd; were separated from their mothers 2 h after birth and were placed into (2.9 m × 1.1 m × 1.8 m; length × width × height) individual pens containing wood sawdust bedding for the duration of the trial. Every day, the pens were refreshed by removing manure and new sawdust was added to make sure that the calves were in dry and clean environments. Physical contact between animals was minimized by using individual pens.

Within 24 h after birth, the calves received 4 L of high-quality (at least 50 g/L IgG concentration) colostrum [52]. This was given in two feedings (< 2 h and < 12 h after birth). Between 24 and 48 h after birth, blood samples were taken from the jugular vein to determine the transfer of passive immunity through measurement of initial serum total protein concentrations (no. T7528, Pointe Scientific, Warsaw, Poland). The concentrations of total serum protein were > 6.0 g/dL for all calves [48], and the difference was not significant (*P* > 0.05). On the 2nd and 3rd days, the calves were given transition milk (4 L/day in 2 equal feedings at 09 h and 17 h). From day 4 until day 49, the calves were given 6 L/day of reconstituted milk replacer in equal amounts three times daily at 06 h, 14 h, and 20 h. From day 50 until day 56 only 2 L milk replacer were offered once daily at 06 h. The 150 g milk replacer powder (23% CP, dry matter (DM) basic, 18% ether extract, and 0.0% crude fiber DM basis (Polmass Red Milk, Bydgoszcz, Poland) were reconstituted with 1 L of water. Throughout the experiment, animals had constant access to fresh water, and water was changed daily. From day 4 onwards, calves were offered pelleted starter feed containing whole corn grain (77/23 w/w, 23% CP, DM basic, Cargill, Kiszkowo, Poland) formulated according to National Research Council guidelines [53] every morning at 10 h ad libitum with an excess of at least 10% (i.e. the amount of the starter, which was not consumed during the last 24 h). Calves were fed the milk replacer and starter feed from the same batch during the complete experimental period. The excess starter feed was collected and weighed daily for each calf. The nutritional composition of the starter feed was analyzed on a weekly basis for 8 representative samples that were collected after the morning feed as described by Stefanska et al., [21]. Procedures of the Association of Official Analytical Chemists [54] were used to analyze the samples for dry matter (method no. 934.01), ether extract ((EE), method no. 973.18), crude protein (method no. 976.05), acid detergent fiber ((ADF), method no. 973.18). The neutral detergent fiber (NDF) was determined by the method described by van Soest et al. [55] and the concentrations of macroelements were measured by inductive emission (ICP-OES) in an Optima 2000 DV Spectrophotometer. The starch content of the starter feeds was determined according to the procedure of Hall [56]. The nutritional and chemical data for the milk replacer and starter feed are shown in Table 5.

**Table 5:** The nutritional value of the milk replacer: and the starter feed (mean ± SD) on a DM basis

Nutritional value (%)^1^	Diet	
	Milk replacer	Starter feed
^2^CP	25.0	23.0 ± 0.16
^3^NDF	-	17.8 ± 0.18
^4^ADF	-	8.10 ± 0.14
Starch	-	43.7 ± 0.35
Ether extract	18.0	2.90 ± 0.12
Ash	6.80	7.00 ± 0.22
Calcium	0.84	0.80 ± 0.08
Phosphorus	0.63	0.58 ± 0.02

^1^The nutritional value of the milk replacer is according to the manufacturer’s information. The representative samples of the starter feed were collected weekly, immediately after the morning delivery, to determine their nutritional value (AOAC: 2010)

^2^*CP* crude protein

^3^*NDF* neutral detergent fiber

^4^*ADF* acid detergent fiber

### Feed intake and growth performance

During the study, calves were weighed on day 3 and then at weekly intervals from week 1 to 8. Individual intake of starter feed was measured daily. For 3 experimental intervals (days 3 to 28, days 29 to 56, and days 3 to 56), average daily gain (calculated as final BW minus the initial BW divided by the number of days), the total dry matter intake (from both the milk replacer and the starter feed), and EF (AGD divided by TDMI) were determined. Individual calf biometric measurements were noted on a weekly basis, starting on day 3. This included BL, HG, HW, and HH as described by Khan et al., [49]. A veterinarian, who was unaware of the animal groupings monitored daily diarrhea and respiratory disease incidences throughout the experimental period. According to the standard operating procedure of the farm, dams were vaccinated twice against rotavirus and coronavirus at approximately day 30 and day 60 before calving. The consistency of faces was recorded every morning, before feeding milk replacer, using the following fecal consistency scoring system: 1 = firm; 2 = soft or of moderate consistency; 3 = runny or mild diarrhea, and 4 = watery and profuse diarrhea [32]. The fecal scores were used for the analysis of the diarrhea incidence; this was done according to recommendations by Liu et al. [20]. Fecal scores ≥ 3 were used as indicative of diarrhea. Calves with diarrhea that lasted for ≥ 24 h were treated orally with electrolytes twice daily (in the morning and evening) using a stomach tube with a manual vacuum pump. The calves received 1 L hydrating dextrose saline solution (glucose 6.23 g/L, sodium chloride 10.7 g/L, sodium carbonate 2.69 g/L, potassium chloride 1.94 g/L). The milk replacer diet was started again when the feces score was 2 or less. During the study, no cases of respiratory disease were noted and no calves died. No antibiotic treatments were administered.

Coproparasitological analyses were performed, to determine the effect of parasites on the health of the calves. Fecal samples were collected from the rectum of calves on days 3, 28, and 56 at about 14 h, which is about 4 h (± 30 min) post provision of the starter feed. The microscopic analyses of the feces were conducted as described by Stefanska et al., [21].

### Ruminal fluid sampling and analysis

On days 28 and 56 at about 14 h, which is about 4 h (± 30 min) post provision of the starter feed, the ruminal content (approximately 150 mL) of each calf was collected using a stomach tube with a manual vacuum pump. To prevent cross-contaminations, the stomach tube was washed with warm water between collections [21]. The first 100 mL of rumen fluid were discarded to minimize saliva contamination. The next 50 mL of ruminal fluid samples were filtered through four layers of cheesecloth into a 500 mL plastic beaker. The ruminal fluid pH was measured using a CP-104 pH meter immediately after sampling (Elmentron, Zabrze, Poland). Samples of ruminal fluid were fractionated into two parts. Part one was used for individual SCFA analyses by gas chromatography (Hewlett-Packard, Waldbronn, Germany) with a flame-ionization detector and Supelco Nukol fused silica capillary column (30 m × 0.25 mm i.d.; 0.25 mm). Part two was used for NH_3_-N concentration measurement analyzed by spectrophotometer (Marcel Media, Zielonka, Poland) as described in detail by Stefanska et al. [21].

### Blood sample collection and analysis

On the first day of the study and then every 14 days throughout the study, blood samples were collected from each calf from the jugular vein at 14 h, which is about 4 h (± 30 min) after provision of the starter feed in the morning. The blood was collected into 10-mL tubes containing polystyrene granules covered with a clotting activator (KABE, Poznan, Poland). The blood tubes were transported to the laboratory, where they were centrifuged at 3000 × g for 15 min at 4 °C to serum obtain. The serum was divided into aliquots and stored at − 20 °C for further analyses of blood urea nitrogen (BUN; no. B7552), and β-hydroxybutyrate (BHBA; no. H7587-58) concentrations using the colorimetric method and Pointe Scientific reagent kits (Warsaw, Poland). The serum samples were diluted initially at a ratio of 1:1 and analyzed in duplicate and absorbance values were read at 450 nm for BUN, and 505 nm for BHBA. Concentrations of non-esterified fatty acids (NEFA) were analyzed according to Duncombe’s colorimetric method (Duncombe, 1964), and absorbance was measured at 440 nm. Serum insulin-like growth factor-I (no. DSL-2800, Diagnostic Systems Laboratories Inc., Webster, TX, USA) was analyzed with a radioimmunoassay method using an Automatic Gamma radiation reader (Gamma Counter 1470, PerkinElmer, Shelton, CT, USA). The inter-and intra-assay variation was controlled by limiting the coefficient of variation to ≤ 5% for all blood variables.

### Statistical analyses

The MIXED procedure within the SAS software version 9.4 [57] was used to analyze the data. The UNIVARIATE procedure of SAS was used to test the normality of the data before any further analyses were carried out. Using a logistic transformation function the fecal score, the total number of parasite oocysts/cysts per gram of feces, and diarrhea occurrence were transformed before statistical analysis. The MIXED procedure was used to analyze the starter intake, growth performance, fecal score, and blood metabolites data for three periods: days 3 to 28; days 29 to 56, and the overall experimental period from days 3 to 56. The mixed procedure used the following model: Y _ijklm_ = µ + l_i_ + m_j_ + p_k_ + t_l_ (p × t)_kl_ + e_ijklm_ where: Y _ijklm_ – is the dependent variable, μ – is the average experimental value, l_i_ – is the random effect of parity of dam (i = is primiparous cows or multiparous cows), m_j_ – is random effect of sex of calf (j = is male or female), p_k_ – is the fixed effect of the measurement period (k = is the number of 14-days measurement periods), t_l_ – is the fixed effect of treatment (l = is CON, MR, SF or MRS treatment), (p × t)_kl_ – is the interaction of period × treatment, and e_ijklm_ – is the error term. In the MIXED MODEL, the fixed effects were period, treatment, and treatment by period interaction and the random effects were dam parity and calf sex. The covariance structures that were tested included CS, Simple, UN, TOEP, AR (1), ARH (1), and ANTE (1) to find the best-fitted structure for the model. A 14-day measurement period was modeled as a repeated measurement by using the compound symmetry as the covariance structure based on best fit, determined by the lowest Bayesian information criterion. The significance of the body weight was determined with an analysis of variance using the SAS PROC GLM, according to the following linear model: Y_ijkl_ = μ + l_i_ + m_j_ + t_k_ + e_ijkl;_ where: Y_ijkl_ – is the value of the analyzed trait; μ- total mean; l_i_ – is the random effect of dam parity (i = is primiparous cows or multiparous cows), m_j_ – is the random effect of sex of calves (j = is male or female); t_k_ – is the fixed effect of treatments (k = CON, MR, SF or MRS); e_ijkl_ – is the random error. Data on EPG, diarrhea occurrences, and ruminal fermentation characteristics were subjected to ANOVA according to the following model: Y_ij_ = µ + Treatment_i_ + e_j,_ where: Y_ij_ – is the dependent variable; μ – is the average experimental value; Treatment_i_ – is the effect of treatment (i = is CON, MR, SF or MRS treatment); e_ij_—is the error term. When differences were detected among treatment or interactions of treatment and period, means separation was conducted using Duncan’s adjustment for the probability. Statistical significance was declared when *P* ≤ 0.05 and trends were indicated when 0.05 < *P* ≤ 0.1.

## Data Availability

All data generated or analyzed during this study are included in this published article.

## Abbreviations

ADF: Acid detergent fiber
ADG: Average daily gain
BHBA: *β*-Hydroxybutyrate
BL: Body length
BUN: Blood urea nitrogen
BW: Body weight
CON: Control group: without eubiotic feed additive in their milk replacer or their starter feed
CP: Crude protein
DM: Dry matter
EE: Ether extract
EPG: Number of parasite oocysts/cysts per gram of feces
FE: Feed efficiency
HG: Heart girth
HH: Hip height
HW: Hip width
IGF-I: Insulin-like growth factor-I
MR: Eubiotic feed additives added to the milk replacer
MRS: Eubiotic feed additives added to the milk replacer and the starter feed
NDF: Neutral detergent fiber
NEFA: Non-esterified fatty acids
N-NH_3_: Ruminal ammonia nitrogen
SCFA: Short-chain fatty acids
SF: Eubiotic feed additives added to the starter feed
TDMI: Total dry matter intake
